# Supplementary material for: A whole-animal phenotypic drug screen identifies suppressors of atherogenic lipoproteins
Source: eLife. 2026 Aug 27;14:RP105314. doi: 10.7554/eLife.105314 (PMC13521566; doi:10.7554/eLife.105314)
Supplement: Supplementary file 2 [file elife-105314-code1.zip › Output_data/JHDL_controls.pdf]

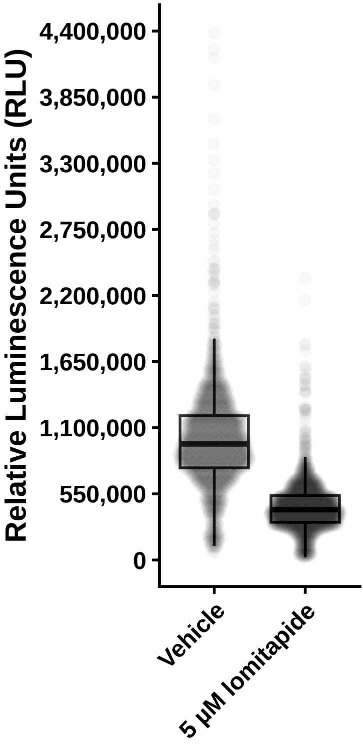
Relative Luminescence Units (RLU)
4,400,000
3,850,000
3,300,000
2,750,000
2,200,000
1,650,000
1,100,000
550,000
0
Vehicle
5 μM lomitapide
